# Volitional down-regulation of the primary auditory cortex via directed attention mediated by real-time fMRI neurofeedback

**DOI:** 10.3934/Neuroscience.2018.3.179

**Published:** 2018-07-07

**Authors:** Matthew S. Sherwood, Jason G. Parker, Emily E. Diller, Subhashini Ganapathy, Kevin Bennett, Jeremy T. Nelson

**Affiliations:** 1 Department of Biomedical, Industrial & Human Factors Engineering, Wright State University, Dayton, OH, USA; 2 Department of Radiology and Imaging Sciences, Indiana University School of Medicine, Indiana University, IN, USA; 3 School of Health Sciences, Purdue University, West Lafayette, IN, USA; 4 Department of Trauma Care, Boonshoft School of Medicine, Wright State University, Dayton, OH, USA; 5 Department of Psychology, Wright State University, Dayton, OH, USA; 6 Department of Defense Hearing Center of Excellence, JBSA-Lackland, USA

**Keywords:** fMRI, neurofeedback, neuromodulation, primary auditory cortex, attention, tinnitus

## Abstract

The present work assessed the efficacy of training volitional down-regulation of the primary auditory cortex (A1) based on real-time functional magnetic resonance imaging neurofeedback (fMRI-NFT). A1 has been shown to be hyperactive in chronic tinnitus patients, and has been implicated as a potential source for the tinnitus percept. 27 healthy volunteers with normal hearing underwent 5 fMRI-NFT sessions: 18 received real neurofeedback and 9 sham neurofeedback. Each session was composed of a simple auditory fMRI followed by 2 runs of A1 fMRI-NFT. The auditory fMRI alternated periods of no auditory with periods of white noise stimulation at 90 dB. A1 activity, defined from a region using the activity during the preceding auditory run, was continuously updated during fMRI-NFT using a simple bar plot, and was accompanied by white noise (90 dB) stimulation for the duration of the scan. Each fMRI-NFT run alternated “relax” periods with “lower” periods. Subjects were instructed to watch the bar during the relax condition and actively reduce the bar by decreasing A1 activation during the lower condition. Average A1 de-activation, representative of the ability to volitionally down-regulate A1, was extracted from each fMRI-NFT run. A1 de-activation was found to increase significantly across training and to be higher in those receiving real neurofeedback. A1 de-activation in sessions 2 and 5 were found to be significantly greater than session 1 in only the group receiving real neurofeedback. The most successful subjects reportedly adopted mindfulness tasks associated with directed attention. For the first time, fMRI-NFT has been applied to teach volitional control of A1 de-activation magnitude over more than 1 session. These are important findings for therapeutic development as the magnitude of A1 activity is altered in tinnitus populations and it is unlikely a single fMRI-NFT session will reverse the effects of tinnitus.

## 1. Introduction

Advances in acquisition techniques, computational power, and algorithms have revolutionized the speed in which functional Magnetic Resonance Imaging (fMRI) data can be measured and processed. This acceleration has led to real-time fMRI, where fMRI data (*i.e.*, blood-oxygen-level-dependent [BOLD] signals) can be processed faster than it is collected. There are currently four domains where real-time fMRI is being implemented: Intraoperative surgical guidance [1], brain-computer interfaces [2],[3], adapting stimuli for current brain states [4], and neurofeedback training [5].

Neurofeedback training (NFT), although not the original focus of real-time fMRI, is a growing field of research where BOLD signals are presented using visual or auditory stimuli during data acquisition so the subject may learn to modulate the signals at will (*i.e.*, closed-loop endogenous neuromodulation). This technique differs from traditional fMRI where individuals respond to exogenous stimuli without being informed of the timing and location of induced brain activity (*i.e.*, open-loop neuromodulation), as well as exogenous neuromodulation techniques like transcranial direct current stimulation (tDCS) or pharmacotherapy. Information regarding the activity of a specific brain region is presented to the subject in real-time during fMRI-NFT. Through the implementation of mental strategies, individuals learn to self-regulate the BOLD signal and, therefore, brain activity as these are tightly coupled through neurovascular mechanisms [6]–[8]. Researchers have shown that people can learn volitional control over the BOLD signal measured from numerous brain regions including the anterior cingulate cortex (ACC) [9], amygdala [10], anterior insula [11],[12], auditory and attention related networks [13], bilateral rostrolateral prefrontal cortex [14], left dorsolateral prefrontal cortex [15]–[17], motor cortices [18]–[20], primary auditory cortex [21]–[23], regions associated with emotional network [24],[25], right inferior frontal gyrus [26], and visual cortices [27],[28].

The efficacy of fMRI-NFT in altering behavior was demonstrated for the first time in 2015 [29]. Since, fMRI-NFT has been demonstrated across a broad range of medical applications. In one study, participants suffering from schizophrenia were able to learn control over the BOLD signal measured from the left and right insula [30]. In another study, an experimental group of participants diagnosed with Parkinson's disease exhibited significant clinical and functional improvements which were not observed from a control group of patients who received sham feedback [31]. Other studies have demonstrated potential applications for people suffering from major depression [32] and chronic tinnitus [21]. One group of researchers combined fMRI-NFT with TMS and found that endogenous neuromodulation of the ventral premotor cortex helps decrease intracortical inhibition measured from TMS. This application may enhance facilitation of stroke victims [18]. Overall, this trend represents the wide range of medical applications of fMRI-NFT but also the specificity of training required for each application.

In several previous studies, control groups who received sham BOLD signals lacked the differences in activity observed from those who received true feedback [9]–[11],[13],[14],[20],[26],[27],[29],[31],[33],[34], implicating neuromodulation or behavioral training strategies affecting global arousal are not effective. Additionally, control groups which received identical instructions and the same period of training but no feedback on the current level of brain activity did not exhibit similar results as the experimental groups who were given neurofeedback [11],[14],[24],[29],[32]. These findings suggest the experiential effects are attributable to real-time fMRI-induced learning rather than other learning or nonspecific changes. Therefore, specific training regimens must be developed which target specific neurophysiological systems to obtain the desired effects. The results from the control groups in Decharms et al. [29] further indicate behavioral training, practice, sensory feedback, and biofeedback alone do not produce effects that are equivalent to those obtained with control groups who receive NFT from real-time fMRI. The exact mechanism translating neuromodulation into behavioral effects are still unknown. One postulation is the brain network responsible for the behavior is reinforced when one actively regulates neural activity in one or more of these regions. Such reinforcement results in the engagement of neuroplastic mechanisms causing the network to execute more efficiently. This theory coincides with other neuromodulation training techniques such as EEG-based neurofeedback where individuals are trained to control frequency bands of electrical signals measured from the local regions of the scalp [35]–[37]. Another hypothesis is that participants who learn appropriate mental strategies to modulate the BOLD signal will recruit task related brain networks more readily than others when processing stimuli [16].

In this work, we investigated the use of fMRI-NFT to teach volitional down-regulation of A1 during binaural auditory stimulation using directed attention strategies. It is not currently known whether individuals are capable of down-regulating A1 in the presence of auditory stimulation. However, two previous studies indicate that volitional down-regulation of A1 activation is achievable. In the one study, twenty-two healthy participants were divided into two equal groups completing the same tasks: one group received neurofeedback and the other group did not. All participants underwent multiple sessions consisting of five neurofeedback blocks. During neurofeedback, participants were asked to increase the activated volume in the primary and secondary auditory cortex from auditory stimulation. The change in activated volume was indicated at the end of each neurofeedback block only for the experimental group. This study found that participants receiving neurofeedback were successful in increasing the activated volume in the primary and secondary auditory cortex using fMRI-NFT; those not receiving neurofeedback were unsuccessful and did not exhibit any trends of habituation to the noise in the fMRI data [23]. In the other study, six participants with chronic tinnitus underwent 4 runs of fMRI-NFT conducted in a single session. It was reported that tinnitus patients were able to volitionally increase A1 activation. However, the study was not controlled so this finding could not be necessarily attributable to fMRI-NFT [21]. The second indicates that fMRI-NFT can train volitional down-regulation. In one uncontrolled study, schizophrenia patients were successfully able to learn volitional down-regulation of the superior temporal gyrus over four fMRI-NFT sessions [38]. In another controlled study, a group of 16 healthy females learned to down-regulate activation of the amygdala in the presence of aversive scenes using one fMRI-NFT session[39]. We used these findings to hypothesize that an experimental group receiving real neurofeedback will have greater volitional control over A1 de-activation than a control group receiving sham feedback.

## 2. Methods

### 2.1. Participants

Healthy volunteers were recruited from Wright State University and the surrounding community. Prior to being enrolled, each potential participant completed a telephone screening to qualify for the study. Forty-seven (47) participants meeting the inclusion/exclusion criteria were recruited (no contraindication to MRI procedures, between the ages of 18 and 50, right handed, no unstable medical or mental illness, no history of neurologic disorders, no hearing loss > 40 dB). These participants were selected at random from the qualifying group. The study was approved by Wright State University's Institutional Review Board (IRB) and the Air Force Medical Support Agency Surgeon General's Research Oversight Committee; informed consent was obtained prior to the execution of any experimental procedures. Participants eligible for compensation received equal remuneration.

Participants were randomly assigned to one of two groups and were blinded to the assigned group. The experimental group (EXP) received real feedback regarding activity in A1 during closed-loop endogenous neuromodulation. The control group (CON) was supplied with sham feedback yoked from a random participant in the experimental group matched for training time. Nineteen (19) participants voluntarily withdrew or were withdrawn from the study due to excessive motion, absenteeism/tardiness, or software/hardware issues limiting the completion of study procedures. The MRI data for a single participant was corrupted. This resulted in a final group of eighteen (18) EXP participants (mean age 23.2 +/− 1.1, 11 males) and nine (9) CON participants (mean age 24.4 +/− 2.5, 4 males).

### 2.2. Experimental design

All participants first completed a consent visit. After obtaining informed consent, the participants completed a MRI screening form. Next, a short hearing test was conducted to verify normal hearing (no frequency > 40 dB on a standard audiogram; Shoebox Audiometry, Ontario, Canada). This test is a simple self-applied test that has been clinically validated [40],[41]. Following the consent visit, the subjects completed five fMRI-NFT sessions. All MRI procedures were conducted on a 3 Tesla (T) MRI (Discovery 750 W, GE Healthcare, Madison, WI) using a 24-channel head coil. These five sessions were executed within 21 days (EXP: 14.61 +/− 0.71 days; CON: 12.44 +/− 1.59) with only one per day.

#### 2.2.1. fMRI-NFT

We performed fMRI-NFT across five sessions for each participant. Prior to entering the MRI environment, MRI screening forms were reviewed by a registered MRI technician. Female participants were required to take a urine dipstick pregnancy test. Once entering the MRI, the participants first inserted MRI-compatible ear plugs (MagnaCoil, Magnacoustics Inc., Atlantic Beach, NY) capable of providing communication and auditory stimulation (Genesis Ultra, Magnacoustics Inc., Atlantic Beach, NY). Next, the participants were positioned supine on the MRI table, their head was padded to restrict motion, and the upper part of the 24-channel head coil was attached. Using a laser, the nasion was landmarked relative to the MRI. The landmarked position was moved to the center of the MRI bore.

**Figure 1.**
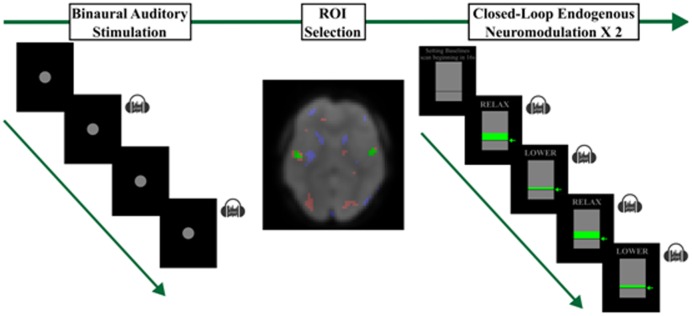
Overview of each fMRI-NFT session. Each session began by acquiring BOLD data during a blocked binaural auditory stimulation paradigm. Next, a region-of-interest for subsequent neurofeedback was selected from activated voxels in the right and left A1. Finally, two runs of closed-loop endogenous neuromodulation were executed to train A1 down-regulation.

Once positioned, the fMRI-NFT procedures began (Figure 1). Each fMRI-NFT session consisted of a single run of bilateral auditory stimulation which was used to individually and functionally localize A1. This scan is referred to as the “functional localizer”. Two runs of closed-loop endogenous neuromodulation followed the functional localizer. A structural MRI was performed between the functional localizer and closed-loop endogenous neuromodulation runs. The structural MRI was acquired using an 3D brain volume imaging (BRAVO) pulse sequence which acquires images using an inversion recovery prepared fast spoiled gradient-echo (FSPGR). The structural images were acquired using a 256 × 256 element matrix, 172 slices oriented in the same plane as the functional scans, 1 mm^3^ isotropic voxels, 0.8 phase field of view factor, TI/TE = 450/3.224 ms, a flip angle of 13°, and an auto-calibrated reconstruction for cartesian sampling with a phase acceleration factor of 2.0. The left and right A1 were manually identified using anatomical markers and an activation map produced from the functional localizer. Once identified, a region-of-interest (ROI) was selected from the voxels in the left and right A1 most robustly activated during the functional localizer. The BOLD signals from this ROI were used to generate the subsequent neurofeedback.

#### 2.2.2. Binaural auditory stimulation

A single run of binaural auditory stimulation was executed to identify A1 using a boxcar design with eight (8) repetitions of OFF and ON blocks. The auditory stimuli were 10 kHz lowpass filtered white noise with a 6 dB roll-off and a 0.5 s fade-in (Audacity 2.1.3, www.audacity.org). The duration of each block was 20 s, and the first block began after the acquisition of four (4) dummy volumes and one (1) software preparation volume. Binaural auditory stimulation was delivered via the headphones only during ON blocks and controlled via a stimulus presentation software (Presentation, Neurobehavioral Systems, Inc., Berkeley, CA). Auditory stimulation consisted of 10 kHz lowpass filtered white noise presented at 90 dB, previously shown to be effective at producing a BOLD response [21]. The participants were not required to respond in any way during the scan, however they were instructed to remain awake and to focus on a round fixation dot presented in gray with a black background on a MRI-compatible display (SensaVue, Invivo, Gainesville, FL). FMRI data were acquired using a gradient-recalled-echo (GRE) sequence sensitive to the BOLD signal. This sequence acquired data using the following parameters: 64 × 64 element matrix, 41 slices oriented parallel to the AC-PC plane, 3.5 × 3.5 × 3 mm^3^ voxels size, 0.5 mm slice gap, TR/TE = 2000/20 ms, and a flip angle of 90° with fat suppression enabled. In previous data collections, these parameters have been shown to reduce susceptibility artifacts which can be significant at high field strengths such as 3T.

#### 2.2.3. ROI selection

Immediately following acquisition, the BOLD data were pre-processed using custom MATLAB and C++ software. The pre-processing included standard spatial filtering (3D, 5-point Gaussian low-pass kernel, full-width half-maximum of 7 mm), motion correction (corrected to the first volume using a rigid-body 3-parameter model) and temporal filtering (5-point Gaussian low-pass kernel, sigma of 3 s) processing functions [42].

An activation map was created by defining a single explanatory variable (EV) by convolving a boxcar model containing 20 s control and task conditions with a pre-defined HRF [43]. Next, the BOLD data at each voxel was fit to the model using a general linear model (GLM) by applying a weight of +1 to the EV, representative of activation (positive correlation to the model). The resulting *β* parameter maps were converted to *t* statistic maps (activation maps) using standard statistical transforms. The region in A1 in which the feedback signal for the subsequent closed-loop endogenous neuromodulation runs was derived from this activation map. Voxels were added to the A1 ROI by first locating the axial slice in which the inferior surface of the anterior ventricle horns is visible. Finally, activation patterns on the left and right hemispheres near the posterior end of the lateral sulci were observed. Voxels within this region responding robustly to binaural auditory stimulation were added to the ROI to complete the determination of the functional localizer.

#### 2.2.4. Closed-loop endogenous neuromodulation

Two runs of closed-loop endogenous neuromodulation were completed following the functional localizer. BOLD data was acquired using the same scan parameters as described for the functional localizer. Four dummy volumes and one software preparation volume were acquired first. Then, eight volumes were acquired to determine a baseline BOLD signal value for the selected A1 ROI. During the acquisition of the baseline volumes, a countdown was displayed on the screen, however there was no auditory stimulation during either the eight baseline volumes or the five preparatory volumes. In the subsequent scanning for the experimental group, a feedback signal was computed and displayed to the participants from real-time analysis of BOLD data. This real-time analysis was implemented in custom MATLAB and C++ software, and included standard spatial filtering (3D, 5-point Gaussian low-pass kernel, full-width half-maximum of 7 mm) and motion-correction (corrected to the first volume of the functional localizer using a rigid-body 3-parameter model) processing functions [42]. This custom software further compared the average BOLD signal in the voxels selected from the functional localizer at baseline to that of the current volume to derive the percent signal change. The current feedback signal was determined by temporally-filtering (5-point Gaussian low-pass kernel consisting of only past components, sigma of 3 s) the percent BOLD signal change with the feedback signals from previous volumes. This feedback signal was presented to the participants using a thermometer-style bar plot within an average of 750 ms from the end of acquisition of a complete volume (∼500 ms for reconstruction and DICOM writing/reading, ∼250 ms for data processing and display). The thermometer plot contained a running average of the previous four values and a running task minimum. For participants in the control group, the feedback signal was yoked from a random EXP participant with experimental progress matched. Both runs from each session were duplicated from the same EXP participant but the EXP participant was selected randomly each session.

After baseline, six repetitions of 30 s relax and lower blocks were completed in a boxcar-design. Both blocks were accompanied with binaural auditory stimulation using the same continuous noise from the functional localizer. During relax, every participant was instructed to relax and clear their mind, resulting in an increase in the feedback signal. They were also instructed to keep their eyes open. Participants were instructed to lower the feedback signal during lower blocks by performing a mindfulness task wherein they should decrease brain activity associated with auditory input. A list of four example mindfulness tasks was provided, giving the participants a few starting points (mindful meditation, thinking about a hobby, doing a mentally engaging task such as math, or thinking about other senses). Through training, participants learned mindfulness tasks that are most successful in regulating A1. Task instructions indicating the current block (relax or lower) were supplied above the thermometer plot.

Participants were then removed from the MRI and escorted out of the MRI room. Participants were then informally interviewed by the experimenter.

### 2.3. Data analysis

The BOLD data acquired from each closed-loop endogenous neuromodulation run was processed using the FMRIB Software Library (FSL) [44],[45] on a 72-core Rocks Cluster Distribution (www.rocksclusters.org) high-performance computing system capable of running 120 threads in parallel. First, individual (first-level) analyses were conducted on each of the 4D fMRI data sets. Prior to the individual analyses, *t* pre-processing was performed using standard techniques. These consisted of applying a high-pass temporal filter (Gaussian-weighted least-squares straight line fitting, cut-off = 60 s) to each voxel, correcting for motion by registering each volume to the center volume of the data set (rigid-body 12-parameter model) [46], creating a brain mask from the first volume and applying to each subsequent volume [47], spatial filtering on each volume using Gaussian convolution (full-width half-maximum of 5.625 mm), and removing low-frequency trends using a local fit of a straight line across time at each voxel with Gaussian weighting within the line to create a smooth response.

Next, individual analyses were conducted on each of the 4D fMRI data sets. A single EV was defined by convolving a boxcar model containing 30 s relax and lower conditions with a HRF (modeled by a gamma function; phase offset = 0 s, standard deviation = 3 s, mean lag = 6 s). The temporal derivative of the original waveform was added to the result. The temporal filter used in pre-processing was applied to the model. The data set was fit to the model using a GLM with prewhitening by applying a weight of −1 to the EV, representative of de-activation during closed-loop endogenous neuromodulation. *Z* statistic maps were created using standard statistical transforms to convert the *β* parameter maps. A clustering method allowed us to account for false positives due to multiple comparisons. This method considers adjacent voxels with a *z* statistic of 2.3 or greater to be a cluster. The significance of each cluster was estimated and compared to a threshold of *p* < 0.05 using Gaussian Random Field theory. The significance of voxels that either did not pass the significance level threshold or do not belong to a cluster were set to zero. A mean image of the data set was registered to the individual's high-resolution structural image by estimating motion from a boundary-based registration method including a fieldmap-based distortion correction [48], then further registered to the MNI-152 T1-weighted 2 mm template provided in FSL [49],[50] using a 12-parameter model. The *z* statistic maps were converted to standard space using the transform responsible for morphing the mean image of each data set to the template to co-register all volumes. A similar process was performed on the BOLD data acquired during the auditory localizer, but a temporal filter with a cut-off of 40 s and a boxcar model with 20 s conditions were used.

#### 2.3.1. ROI-based analysis

The target ROI coordinates in each fMRI-NFT session were converted to a binary mask. Since the ROI was determined from the first volume of the functional localizer, motion was corrected in the functional localizer data by registering each volume to the first volume using the method described above and a mean image was created. Next, the mean image of each neuromodulation run was registered to the mean image of the associated functional localizer using a rigid-body 12-parameter model. The transform responsible for morphing the mean image of each neuromodulation run was applied to the associated ROI mask. Volitional down-regulation of A1 was assessed in both groups by masking the de-activation map (*i.e.*, decreased BOLD signal during the lower condition compared with the relax condition) from above with the registered ROI mask. A mixed factor ANOVA (between-subjects factor: Group; within-subjects factors: Session and run) was performed on the neuromodulation performance metric using SPSS (IBM SPSS statistics version 24.0, IBM Corp., Amonk, New York).

#### 2.3.2. Whole brain analysis

Group (second level) analyses were performed in FSL using to conduct a voxel-wise 2 × 2 (between subject factor: Group; within-subjects factor: Session) mixed factor ANOVA in FSL. Run 1 from the first fMRI-NFT session and run 2 from the last fMRI-NFT session were included to assess the overall change in A1 de-activation. Prior to running this analysis, each individual de-activation map was masked to remove activated voxels. This enabled us to assess only changes in de-activation, as the results of the ANOVA are bi-directional. The 2 × 2 ANOVA analysis assumed the covariance between measures within-subject follow a compound symmetric structure (equal variance and intra-subject correlations being equal). This assumption is valid as the data was acquired in close proximity and regularly sampled. Two contrasts were created to identify voxels more de-active during the fifth training session than the first session and a larger change in de-activation from the first to fifth training session (5–1) for the EXP group than the CON group. *Z* statistic maps, created by transforming the resulting *β* parameter maps using standard statistical transforms, were thresholded using the clustering method outlined above with a *z* statistic threshold of 1.96. Furthermore, *β* parameter estimates from each of these contrasts underwent separate *F* tests to explore the main effect of session and the session by group interaction. This analysis lacked the degrees of freedom necessary to include the main effect of group and, therefore, this contrast was not included. *Z* statistic images were created from *F* statistic images using standard statistical transformations. This group analysis was repeated using the auditory localizer from sessions 1 and 5.

## 3. Results

Using an independent samples *t*-test, the mean age for each the EXP group was found to not significantly differ from the CON group (*t*_25_ = 1.447, *p* = 0.160, two-tailed). Equal variances were assumed as Levene's test was not significant (F_1.25_ = 3.832, *p* = 0.062). Furthermore, the training time, calculated as the separation between the first and last neurofeedback session, did not significantly differ between the EXP and CON groups (*t*_25_ = −0.522, *p* = 0.606, two-tailed). Equal variances were assumed as Levene's test was not significant (F_1.25_ = 1.278, *p* = 0.269).

### 3.1. ROI-based analysis

**Figure 2.**
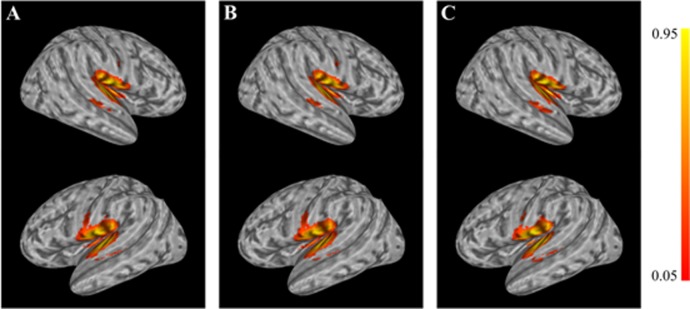
The probability of voxel inclusion during fMRI-NFT for: (A) both EXP and CON groups; (B) EXP group only; (C) CON group only. ROIs were transformed to standard space using the same transformation responsible for morphing the fMRI data to standard space. Yellow voxels were included most frequently in the functionally-defined ROIs while red voxels were selected less frequently.

A mixed factor ANOVA evaluated the size (overall average = 1490 mm^3^) of the functionally-defined ROI across sessions and groups (Figure 2). The size of the ROIs did not significantly differ between sessions or groups (*p* = 0.567, *p* = 0.108, respectively, two-tailed). Furthermore, the interaction of session by group was not significant in the ROI size (*p* = 0.713, two-tailed). Although the ROIs for the CON group were not used during neurofeedback, these ROIs were utilized for post-processing to compute A1 de-activation. The average size of each ROI across groups and sessions was 1490 mm^3^ ± 283.15 mm^3^. Furthermore, the 3D coordinates of the center of mass (COM) or each ROI was computed from the standard-space transformed ROI per session and hemisphere. A total of six 5 × 2 mixed factor ANOVAs evaluated the COM location for each dimension and hemisphere across sessions and groups. The main effect of session was significant for only the z-dimension in the right hemisphere (F_4,100_ = 5.098, *p* = 0.001). Bonferroni-corrected *post hoc* comparisons revealed the z-location at session 2 varied significantly from sessions 1 and 4 (*p* = 0.003 and *p* = 0.002, respectively). The main effect of session was not significant for any of the other dimensions/hemispheres (*p* > 0.05). The main effects of group and the group by session interactions were not significant (*p* > 0.05) for all three dimensions and both hemispheres, suggesting the location of the selected ROIs were consistent across groups and the small variation in the right z-dimension did not vary differently between groups.

**Figure 3.**
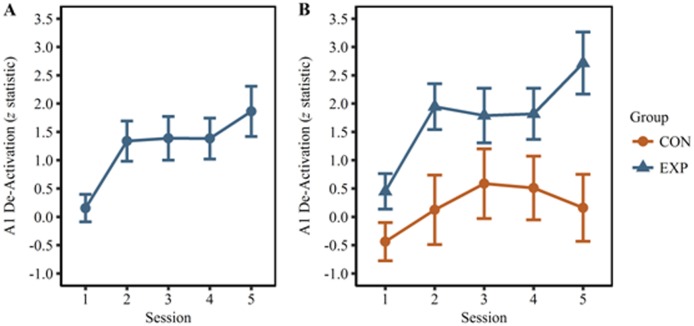
A1 de-activation during closed-loop endogenous neuromodulation. (A) A1 de-activation averaged across groups and runs for each session. The main effect of session was found to be significant (*p* = 0.0175). (B) A1 de-activation averaged across runs separated by group and session. The *post hoc* pairwise comparisons did not reveal any significant differences for the CON group, however sessions 2 (*p* = 0.038) and 5 (*p* = 0.0165) were found to be significantly greater than session 1 for the EXP group.

The effects of group, session, and run on A1 de-activation during closed-loop endogenous neuromodulation were evaluated using a mixed factor ANOVA. A1 de-activation is representative of an individual's ability to volitionally down-regulate A1. The results of the tests of between-subjects effects (Table 1) revealed a significant main effect of group (*p* = 0.029, one-tailed). One-tailed statistics are reported (the a priori hypothesis was that A1 de-activation would be greater in the EXP group). The ANOVA analysis included Mauchly's Test of Sphericity which determined that the variances of the differences between all possible pairs of within-subject conditions were not significant for the main effect of session (*p* = 0.160, two-tailed) or the interaction of session and run (*p* = 0.776, two-tailed). This test could not be conducted on the main effect of run because there is only a single difference to compute and, therefore, no comparison to be made. These results validate the assumption of sphericity, which was used to assess the results of the within-subjects tests henceforth. The results of the within-subjects testing (Table 2) identify a significant main effect of session (Figure 3; *p* = 0.0175, one-tailed). One-tailed statistics are reported (the a priori hypothesis was that A1 de-activation would increase with training). The main effect of run was not significant (*p* = 0.283, one-tailed). The interaction effects of session by group, run by group, session by run, and session by group and run were not significant (*p* = 0.225, *p* = 0.175, *p* = 0.070, and *p* = 0.218, respectively).

**Table 1.** A1 de-activation ANOVA between-subjects test results. Power was computed using an alpha of 0.05. Highlighted rows indicate significance at or below *p* = 0.05.

Source	Type III Sum of Squares	df	Mean Square	F	Sig. (one-tailed)	Partial Eta Squared	Observed Power
Intercept	22.381	1	22.381	6.073	0.011	0.195	0.659
Group	14.524	1	14.524	3.941	0.029	0.136	0.480
Error	92.135	25	3.685				

**Table 2.** A1 de-activation ANOVA within-subjects test results. Power was computed using an alpha of 0.05. Highlighted rows indicate significance at or below *p* = 0.05.

Factor	Type III Sum of Squares	df	Mean Square	F	Sig. (one-tailed)	Partial Eta Squared	Observed Power
Session	59.395	4	14.849	2.702	0.0175	0.098	0.731
Session* Group	20.447	4	5.112	0.930	0.225	0.036	0.286
Run	0.933	1	0.933	0.338	0.283	0.013	0.087
Run* Group	2.506	1	2.506	0.908	0.175	0.035	0.150
Session* Run	11.377	4	2.844	1.772	0.070	0.066	0.524
Session* Run* Group	6.121	4	1.530	0.953	0.218	0.037	0.292

Post hoc, Bonferroni-corrected pairwise comparisons were conducted on the session by group interaction. These results revealed no significant difference between session 1 and 5 for the CON group (*p* > 0.05; Table 3); however, a significant difference between these sessions was identified in the EXP group (*p* = 0.0165, one-tailed). There was also a significant difference between sessions 1 and 2 for the EXP group (*p* = 0.038, one-tailed). Furthermore, the EXP group was found to have significantly greater A1 de-activation than the CON groups on session 2 (*p* = 0.031, one-tailed) and 5 (*p* = 0.021, one-tailed) as identified in Table 4.

**Table 3.** A1 de-activation *post hoc* pairwise comparison results for session by group. Statistical significances were computed using Bonferroni correction for multiple comparisons. Highlighted rows indicate significance at or below *p* = 0.05. The reported statistical significance is one-tailed due to the *a priori* hypotheses.

Group	(I) Session	(J) Session	Mean Difference (I–J)	Std. Error	Sig. (one-tailed)
CON	1	2	−0.563	0.730	1.000
		3	−1.024	0.802	1.000
		4	−0.949	0.813	1.000
		5	−0.597	0.978	1.000
	2	3	−0.461	0.719	1.000
		4	−0.386	0.828	1.000
		5	−0.034	0.829	1.000
	3	4	0.076	0.742	1.000
		5	0.427	0.739	1.000
	4	5	0.352	0.571	1.000
EXP	1	2	−1.496	0.516	0.038
		3	−1.339	0.567	0.132
		4	−1.369	0.575	0.125
		5	−2.266	0.692	0.0165
	2	3	0.157	0.509	1.000
		4	0.126	0.586	1.000
		5	−0.770	0.586	1.000
	3	4	−0.030	0.525	1.000
		5	−0.927	0.523	0.441
	4	5	−0.897	0.404	0.178

**Table 4.** A1 de-activation *post hoc* pairwise comparison results for group by session. Statistical significances were computed using Bonferroni correction for multiple comparisons. Highlighted rows indicate significance at or below *p* = 0.05. The reported statistical significance is one-tailed due to the *a priori* hypotheses.

Session	(I) Group	(J) Group	Mean Difference (I–J)	Std. Error	Sig. (one-tailed)
1	EXP	CON	0.888	0.585	0.071
2	EXP	CON	1.821	0.931	0.031
3	EXP	CON	1.203	1.121	0.147
4	EXP	CON	1.309	1.007	0.103
5	EXP	CON	2.557	1.194	0.021

### 3.2. Whole brain analysis

**Figure 4.**
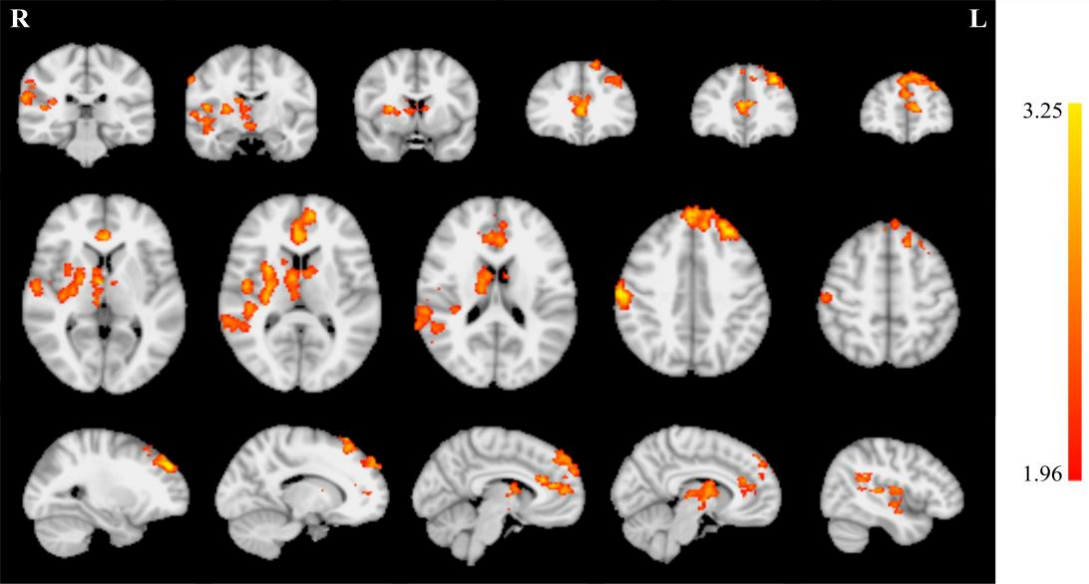
*F* test results for the main effect of session indicate increased de-activation during neurofeedback across training for several brain regions. Coronal slices (top row) are displayed at MNI coordinates y = −30, −10, 6, 34, 40 and 52 mm (left to right). Axial slices (middle row) are displayed at MNI coordinates z = 4, 10, 18, 40, and 52 mm (left to right). Sagittal slices (bottom row) are displayed at MNI coordinates y = −26, −14, −6, 8, and 44 mm (left to right).

A 2 × 2 (group by session) mixed factor ANOVA was performed on the BOLD data from the session 1 run 1 and session 5 run 2 closed-loop endogenous neuromodulation runs using FSL. The *F* test revealed a significant (z > 1.96) main effect of session on de-activation magnitude during closed-loop endogenous neuromodulation in several regions throughout the brain (Figure 4; Table 5). Increased de-activation was observed across training in auditory regions (superior temporal gyrus, transverse temporal gyrus, and insula) limited to the right hemisphere. In contrast, increased de-activation in attention-related regions (medial frontal gyrus, superior frontal gyrus, and middle frontal gyrus) were observed in the left hemisphere. However, bilateral changes in the anterior cingulate and caudate were observed.

**Table 5.** Local maxima for the *F* test results for the main effect of session. Coordinates are specified in MNI space.

	Coordinates		
*Z* statistic	X (mm)	Y (mm)	Z (mm)
3.82	62	−16	42
3.55	−26	46	38
3.4	−32	40	40
3.36	54	−42	24
3.33	2	38	12
3.3	60	−36	24
3.22	4	54	40
3.15	30	−20	12
3.15	0	34	6
3.14	28	6	10
3.14	−40	28	22

**Figure 5.**
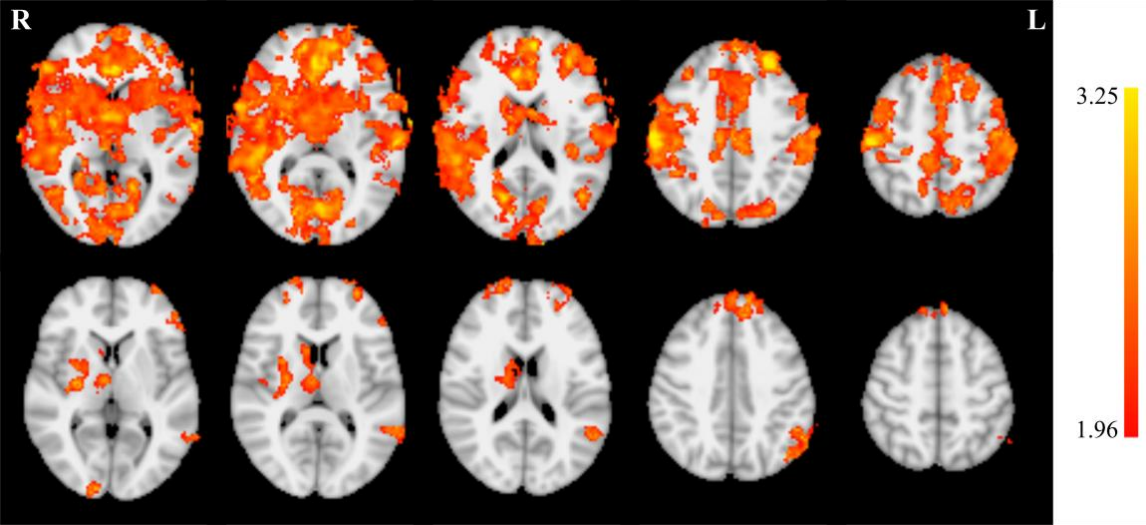
Increasing de-activation across training by group. The EXP group (top row) demonstrated a larger increase in de-activation across training than the CON group (bottom row) in both magnitude and extent. Axial slices (middle row) are displayed at MNI coordinates z = 4, 10, 18, 40, and 52 mm (left to right).

Additional pairwise comparisons revealed a large increase in de-activation magnitude for the EXP group (Figure 5, top row; Table 6). This was apparent in both magnitude and extent. Increases in de-activation magnitude were also observed in the CON group (Figure 5, bottom row; Table 7), however these effects were smaller and more focal than in the EXP group.

**Table 6.** Local maxima for increased de-activation across training for the EXP group. Coordinates are specified in MNI space.

	Coordinates		
*Z* statistic	X (mm)	Y (mm)	Z (mm)
4.31	10	−86	4
3.97	62	−18	42
3.86	−66	−12	4
3.85	34	18	−22
3.83	−14	104	18
3.74	−12	−58	68

**Figure 6.**
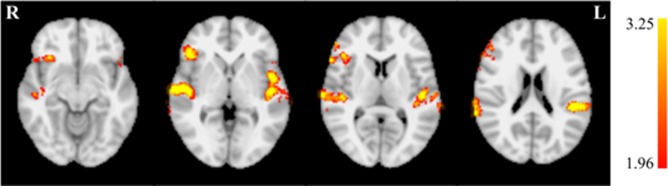
*F* test results for the main effect of session indicate decreased activation during auditory stimulation across training bilaterally in the superior temporal gyrus and in the right inferior frontal gyrus. Axial slices are displayed at MNI coordinates y = −10, 0, 8 and 20 mm (left to right).

An additional 2 × 2 (group by session) mixed factor ANOVA was performed on the BOLD data using the session 1 and 5 auditory localizer using FSL. The *F* test revealed a significant (z > 1.96) main effect of session on activation magnitude during auditory stimulation in the right inferior frontal gyrus and bilaterally in the superior temporal gyrus (Figure 6). The contrast identifying voxels with significantly greater activation in session 1 compared to session 5 was assessed to clarify the directionality. This contrast identified the regions in the right inferior frontal gyrus and bilateral superior temporal gyrus, implying activation in these regions significantly decreased with training. However, there were no significant findings in the *F* test for the session by group interaction and, thus, these changes were not found to vary significantly between groups and, thus, suggests the differences in neurofeedback performance cannot be explained by variations in the auditory localizer.

**Table 7.** Cluster maxima for increased de-activation across training for the CON group. Coordinates are specified in MNI space.

	Coordinates		
*Z* statistic	X (mm)	Y (mm)	Z (mm)
3.45	−45	−54	28
3.4	−56	−32	−16
3.33	28	−12	8
3.23	−20	64	24
3.23	−52	46	−10
3.17	22	−98	−8

## 4. Discussion

Training self-regulation of brain activity from fMRI-NFT has shown promise in a broad range of applications such as the improvement of human performance [15],[16],[27] and a variety of medical applications including recovery from stroke [18],[20], major depression [32],[51], Parkinson's disease [31], and chronic pain [29]. Of the techniques currently being explored, endogenous neuromodulation techniques [6],[44],[52] have the advantages of no known side effects and may be translated to exercises that could be performed at home without the use of sophisticated equipment and trained professionals [11],[32]. Real-time functional magnetic resonance imaging [53],[54] has seen a dramatic rise in interest since its advent in 1995, with a large portion of research dedicated to its application for training endogenous neuromodulation. In this technique, termed closed-loop endogenous neuromodulation, the BOLD signal is measured from a specific region of the brain, processed, and presented to the subject in real-time. Through training, subjects develop self-directed mental processing techniques that regulate this signal.

The present study found evidence for successful down-regulation of A1 using fMRI-NFT. The experimental group attempted down-regulation with the aid of real information regarding the current BOLD signals in A1 while the control group was supplied sham feedback yoked from a random participant in the experimental group and matched for training progress. In both groups, the bilateral A1 was identified both anatomically and functionally using an activation map produced during binaural continuous noise stimulation at each of the five training sessions. The results indicate an overall increase in the ability to volitionally decrease A1 activity across training. The most successful participants reported focusing on breathing during “lower” conditions during neurofeedback. A1 de-activation was not found to be significantly different at the first session between the experimental and control groups. However, the ability to volitionally decrease A1 activity was observed to be significantly greater for the experimental group compared to the control group at sessions two and five. Furthermore, self-control over A1 de-activation between the first and last training session was significantly increased in the experimental group. There was also a significant increase between the first and second training session signifying a rapid effect of neurofeedback training on A1 de-activation. These effects were not observed in the control group.

Interestingly, attempting volitional down-regulation of the auditory cortex resulted in a right-lateralized increase of de-activation in the occipital cortex (Figure 3). Asymmetry in the auditory system is well-documented (*i.e.*, “right ear advantage”), thought to be largely due to language processing regions contained in the left hemisphere. Typically, right ear advantage would be observed in the left hemisphere, as the sensory tracts largely decussate so that right ear information is almost entirely processed in the left hemisphere. However, due to the assessment of de-activation in the present study, the right ear advantage might reflect the right-lateralized results. There is a potential for handedness to also play a role similar to the right ear advantage, with similar logic regarding results apparent in de-activation. Additionally, habituation to the noise could be a contributing factor, though unlikely as habituation would have manifested as decreased activation in auditory regions equally apparent in both lower and relax conditions of the neurofeedback training. However, since this result arouse in both groups on average (main effect of session), our study lacks the control group necessary to disregard the potential of these effects as a result from habituation.

Our results add to a growing body of research that demonstrates the success of fMRI-NFT in teaching individuals to self-regulate localized brain activity. A previous controlled study indicates healthy individuals can learn to control the activated cortical volume in the primary and secondary auditory cortex using fMRI-NFT [23]. A second previous study indicated that control over the magnitude of A1 activation is also achievable however not necessarily attributable to fMRI-NFT [21]. The results above add to these previous studies by indicating fMRI-NFT aids control over the magnitude of A1 de-activation. In addition, this result shows that 60 min of distributed fMRI-NFT is adequate to train volitional A1 down-regulation, but significant observable effects are prevalent after only 24 min of training.

Our findings are important in the search for a possible treatment and/or therapy for tinnitus. Tinnitus, the phantom perception of sound, is often a symptom of an underlying condition such as age-related hearing loss, ear injury, or a circulatory system disorder. The phantom noise is highly variable in laterality, pulsatility, percept (pitch, intensity), and duration. Furthermore, a central mechanism has been implied as the percept remains following the complete resection of the auditory nerve [55] and acoustic tumors [56]. Tinnitus has been associated with hyperactivity in the auditory cortex in response to auditory stimulation [57],[58] and at rest [59]–[62]. Additionally, altered attentional processes has been implicated as the source of the percept [58],[63]. Only one previous study has investigated fMRI-NFT as a possible treatment for tinnitus [21]. In their study, four 4 min closed-loop endogenous neuromodulation runs to train up-regulation of A1 activation were completed in a single training session. The behavioral assessments were conducted before and after the single fMRI-NFT session. Their study indicates the promise of fMRI-NFT in treating tinnitus, but only included six participants and did not offer a control group. Furthermore, the researchers did not perform any statistical analysis on the behavioral data. Our study is unique for two reasons. First, we employed fMRI-NFT to reduce A1 activity which, as the literature suggests, is hyperactive in tinnitus populations. Second, A1 activity was reduced by directing attention away from the auditory cortex. This is important since it has been suggested that over-attention is drawn toward auditory processing in tinnitus populations.

## 5. Conclusion

The results presented in this work align with previous findings which indicate fMRI-NFT can be used to teach participants to voluntarily control the auditory cortex. However, the results of the presented work add to the previous findings by indicating volitional down-regulation of the auditory cortex is achievable in the presence of continuous noise using fMRI neurofeedback. This has not been previously reported. Tinnitus can cause severe impairments and can even limit the ability to perform daily functions. The financial burden associated with tinnitus is extensive. The number of U.S. veterans receiving service-connected disability for tinnitus exceeded all other disorders including post-traumatic stress disorder, hearing loss, and major depression [Annual Benefits Report Fiscal Year 2014, U.S. Department of Veterans Affairs]. The tinnitus percept is attributed to a central mechanism. Also, tinnitus has been associated with hyperactivity in the auditory cortex and abnormal attentional processes (theorized to cause the tinnitus percept). The results presented suggest attempting down-regulation of the auditory cortex may be a possible treatment for tinnitus by decreasing hyperactivity of the primary auditory cortex and directing attention away from auditory processing. Future work is necessary to study these procedures in a cohort of tinnitus patients but should also assess changes in activation associated with volitional down-regulation of the auditory cortex.
